# Supplementary material for: Short-Term Effect of SARS-CoV-2 Spike Protein Receptor-Binding Domain-Specific Antibody Induction on Neutrophil-Mediated Immune Response in Mice
Source: Int J Mol Sci. 2022 Jul 26;23(15):8234. doi: 10.3390/ijms23158234 (PMC9331224; doi:10.3390/ijms23158234)
Supplement: Supplementary file 1 [file ijms-23-08234-s001.zip › ijms-1820121-supplementary.pdf]

Supplementary Figures

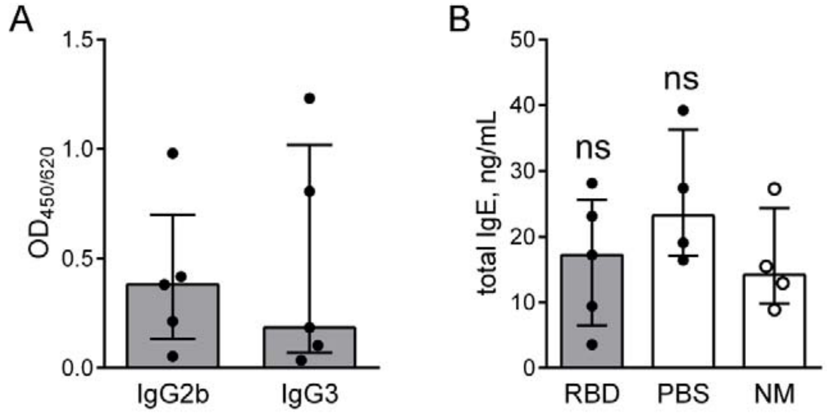

Supplementary Figure S1. Peripheral blood serum antibodies in the RBD-injected mice. A. The levels of periphery blood RBD-specific IgG2b (left barF) and IgG3 (right bar) a week after the last injection, serum dilution 1:1000. B. Total IgE concentration in the peripheral blood serum of the RBD- (gray bars, black circles) or PBS-injected (open bars, black circles), and in the intact mice (NM, open bars, open circles) a week after the last injection, serum dilution 1:100. The representative data are shown (n≥4 mice per group). Significant difference between the indicated group and the intact mice was detected us-ing Mann–Whitney test ns: $p > 0.05$.

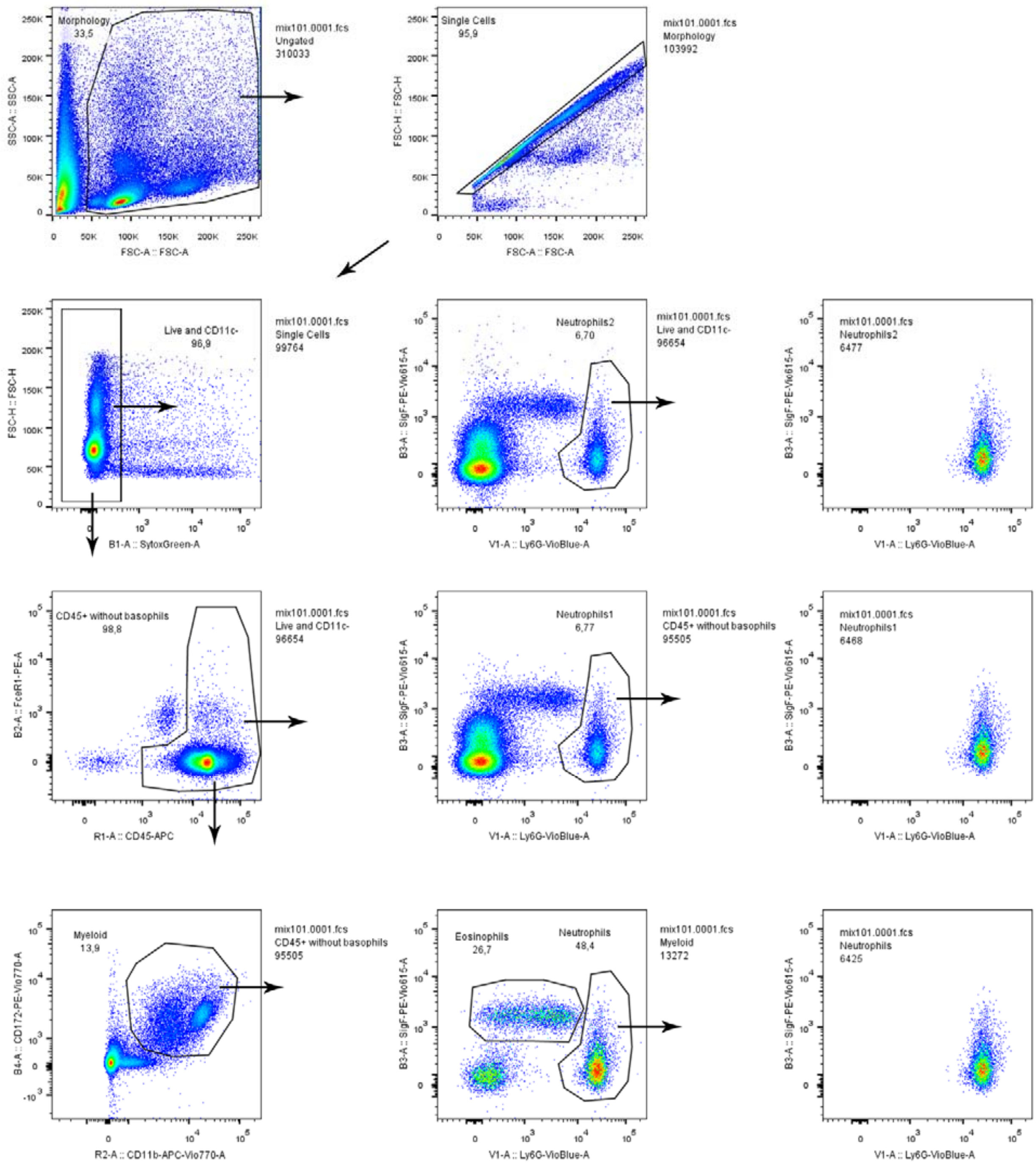

Supplementary Figure S2. Neutrophil gating strategy.

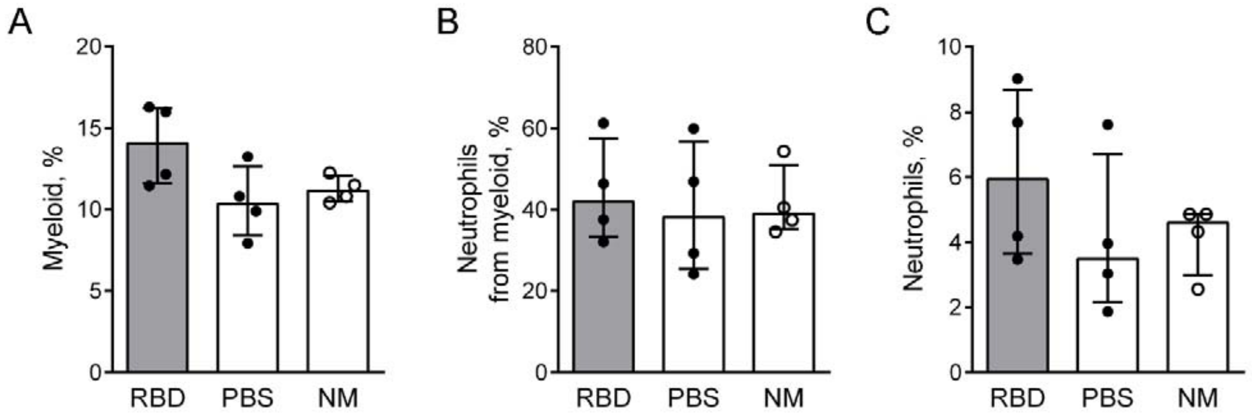

Supplementary Figure S3. The effect of RBD-injections on the neutrophil recruitment to the blood. The percentages of myeloid cells (A), neutrophils from myeloid cells (B) and neutrophils from live blood cells (C) a week after the i.p. injections of RBD (gray bars, black circles) or PBS (open bars, black circles); the intact mice NM (open bars, open circles). The data are shown as medians and interquartile range (i.q.r.) for n=4 mice per group. Significant difference between the indicated group and the intact mice was detected using the Mann–Whitney test.
